# People's Restaurants

**Published:** 1901-01-12

**Authors:** 


### THE INSTITUTIONAL WORKSHOP.

### PEOPLE'S RESTAURANTS.

As civilisation advances, and the sense of responsibility in the individual for his fellows in cities increases, the desire to secure adequate arrangements for feeding the people increases too. Indeed in nations where the rush and scramble of modern life is less felt than in countries where commercial enterprise has quickened the desire in all classes to grow rapidly prosperous, and where, in consequence, there is more time at the disposal of everybody to think out problems which do not directly concern the business or lives of the more prosperous classes, there the problem of feeding the poorer residents in cities has been solved to a greater extent and on a sounder basis than elsewhere. Thus, the Norwegians have an excellent system at Christiania whereby every poor family can obtain sound, wholesome food, well cooked and palatable, at very small cost. Naturally the same system prevails in Sweden, and in the city of Vienna there is probably the most complete system of people's restaurants to be found anywhere. We have visited all these countries, have enquired very closely into the systems pursued and have been struck with the popularity of the provision made and its success, both financially and generally. That success is due to an appreciation of the habits and tastes of the various peoples for whom the provision has been made; and it will surprise nobody to hear that the kinds of food and methods of cooking, and the various articles which are most popular at the people's restaurants referred to, differ very materially in Norway and Sweden, and to a greater extent still in Austria, when a comparison is made between the Viennese plan and that pursued in the northern countries referred to. Still, the encouraging fact, and one which has constantly to be borne in mind, is that all three systems have proved successful, and that their success is increasing year by year.

### INERTNESS OF THE PROSPEROUS CLASSES.

To state this is to condemn the inertness of the more prosperous classes in the great English towns and cities, where practically no attempt has been made to enable the poorer citizens to procure the food they need in adequate quantity, properly cooked and served, under conditions suitable to their habits and tastes. Of course, the A B C, Lyons, and Lockhart systems, managed as commercial undertakings and earning good dividends for the proprietors, have met a want in recent years which was formerly greatly felt by the middle and working classes who patronise these establishments throughout London. None of these systems attempt, however, to do for the humbler citizens what has been accomplished by independent organisations in Norway, Sweden, and Austria. Yet the enormous waste which goes on in a great city like London, a waste which could be largely prevented by the wise utilisation of much wholesome food which is now thrown away, is a reflection upon English methods of living, and should be a just cause for keen criticism on the part of the intelligent foreigner. There is no reason, however—quite the contrary—why a business people like the English should permit this waste to continue, for the enormous fortunes which have been made by enterprising publishing firms which have sprung up within the last ten years, and which have depended mainly on the lower and poorer classes for their prosperity, show that under proper management it is far more profitable as a matter of business to cater for the multitude than to invest one's capital in enterprises which provide only for the relatively few and prosperous. In such circumstances it is the more surprising that the first real attempt to set up a people's restaurant in London, of the kind we refer to, should have its origin, not in any desire to provide cheap food for the multitude as a matter of business, but purely as a matter of sympathy.

### THE ALEXANDRA TRUST.

It will be remembered that at the time of the Diamond Jubilee H.R.H. the Princess of Wales, who is probably the most sympathetic and kind-hearted of all the subjects of the Queen, felt that women ought to take some step which should make the celebration of Her Majesty's Diamond Jubilee a day of joyousness for the poorest of the poor. Her Royal Highness accordingly wrote a letter expressing the hope that arrangements would be made, and money would be forthcoming, to provide a banquet for this class of Londoners upon a plan which should secure that everybody, however miserable and however poor, should obtain admission to it, and so have a good meal and real enjoyment for that day at least. The cost, £25,000, was defrayed by a cheque in response to Her Royal Highness's appeal, presented by Sir Thomas Lipton, who sent it to the Mansion House, and so enabled Sir Faudel and Lady Phillips to give practical expression to the idea formulated by the Princess of Wales which proved in practice entirely successful. Sir Thomas Lipton, having put his hand to the plough, after full consideration determined to go a step further in a direction which had always attracted him, and which, as a young man, he had made up his mind should have his utmost support should he ever attain to a position of prosperity and influence. In the result he gave a quarter of a million of money to establish the Alexandra Trust, of which H.R.H. the Princess of Wales is President, and invested the money in the names of certain trustees, for the purpose of providing suitable dining rooms and kitchens, where cheap meals of the most varied and best kind could be supplied, at the minimum cost, to all poor people who cared to resort to them. Sir Thomas Lipton then set to work with his usual energy to design and erect a really magnificent restaurant and dining rooms in the City Road upon a freehold site, which contains accommodation for 1,500 guests.

### A PERFECTED ORGANISATION.

We visited the Alexandra Trust dining rooms recently, without any notice, and were immensely struck with the evident good which was being done to a multitude of poor people through its agency, and especially by the excellent administration of the whole establishment. The kitchens are at the top of the building, and here dinners and other meals are prepared and supplied to some 3,000 people every day during six days in the week. We timed our visit so as to arrive between one and half-past, the busiest time of the day, and on visiting the kitchens were much impressed with the cleanliness, the good order, and the quiet rapidity which were everywhere apparent, and which made it possible to serve 1,500 dinners in a very few minutes, every dinner being excellent of its kind, hot, tempting, good in quality, and at a price which is within the means of everybody, however poor. The patrons of the Alexandra Trust can procure a three-course dinner, consisting of soup, meat, two vegetables, and pastry, or pudding, or, instead of the last named item, a cup of tea, coffee, or cocoa, for 4½d. A large variety of vegetables is given, including cabbage, turnips, potatoes cooked in various ways, haricot beans, and parsnips. Of the meats the best demand is for steak puddings: these are so popular that they have to be kept on the bill of fare every day; and next to them in popularity come roast beef and roast mutton. These dining rooms are opened at 6.30 a.m., the busiest time of the day being the dinner hours from 12 to 2, the number of guests reaching the maximum between one and half-past, when, as a rule, the dining rooms are crowded to their fullest extent. The dining rooms close at 8 o'clock; and they are frequently full between 4 and 5, when a large number of people frequent them for tea. In addition to the meals served to the guests in the dining rooms, arrangements have been made whereby anything on the bill of fare can be purchased and taken away to the people's homes. This outside system of supply is not yet fully realised or developed, but after six months' working from 150 to 200 meals are sent out of the establishment each day. Down to October 31st, 1900, that is, during the eight months during which the dining rooms have been open, some 569,449 meals have been served, or a daily average of 2,861. The three-course dinner represents more than one-third of the whole number of meals served, and next to this course come the teas which amount to nearly the same number, showing, we infer, that the 2,000 people who have ordered them are regular customers each day at the Alexandra Trust dining-rooms for dinner and tea. Mondays and Saturdays compare unfavourably with the other four days of the week; a fact which illustrates the life and habits of the working and poorer classes in London at the present time, but on the other four days of each week the average number of guests has been 3,300. It is interesting to notice that the number of customers served in July exceeded that of any month, owing, it is believed, to the special efforts which were made to supply suitable meals for the hot weather. However, as the dining rooms have become known, the number of those who partake of the three-course dinner has so largely increased that the takings in the month of October exceeded those in July by £70.

### An Ideal Out-Patient Refreshment Bar.

Every effort is being made to cater for the different tastes of those using the dining-rooms. A special pot of tea can be had for 1½d. and a plate of bread and butter for 1d. The first floor of the restaurant is reserved for this new feature of the Trust after 3 p.m., and is being increasingly patronised week by week. Morning and evening newspapers are now provided for the use of the customers, who much appreciate this attention. The dining rooms are in the immediate neighbourhood of the Royal London Ophthalmic Hospital, and the Alexandra Trust has opened a refreshment bar at this hospital for the convenience of the out-patients. Since this step was taken upwards of 5,000 persons have availed themselves of the opportunity afforded them to purchase food at this refreshment bar. The trustees are in communication with the authorities of the London School Board with a view to the supply of meals to Board School children, but it has not yet been found possible to make any definite arrangements in this direction. When we visited the dining-rooms we noticed, however, that several of the tables, many of which are purposely small, were occupied by family groups, five or six children taking their meals with their parents, and all being evidently very happy and comfortable.

### An Extended System of People's Restaurants.

We are of opinion that London owes a considerable debt of gratitute to H.R.H. the Princess of Wales and Sir Thomas Lipton for the thought, time, energy, and money which have been expended upon this excellent movement, so full of promise and of hope for the poorer class of citizens. It seems to us that the Alexandra Trust dining-rooms, which are of magnificent proportions and considerable architectural pretensions, may usefully be made the centre from which a large number of smaller establishments under the direction of the Trust can be provided with food and other supplies requisite to their successful opening and maintenance. There can be no question that, with the guarantee which the energy and administrative ability of Sir Thomas Lipton supply, an extended system of people's restaurants of a smaller and less pretentious kind, though equally well managed and supplied with every requisite for feeding the people should they be opened in every part of London where the working classes reside, would immeasurably benefit directly the poor and least well-to-do, and indirectly the whole metropolis of the Empire, by making the lives of the majority much more healthy and much more enjoyable than they are at present. It is a great satisfaction to us to learn that Sir Thomas Lipton intends to extend his Alexandra Trust system of dining-rooms in this way, and we can only hope that as little time as possible will be lost in developing this movement, so that it may be happily introduced into every part of London. If this is once done, we are confident, from what we have seen abroad, and from a careful study of the working and results of the Alexandra Trust dining-rooms in the City Road, that before many years are past every large city in the United Kingdom will adopt some such system as that we have here described, which the nation owes to the perspicuity of H.R.H. the Princess of Wales and the liberality and public spirit of Sir Thomas Lipton.

### Economical Aspects.

It has been maintained by English contractors that the actual rate of wages paid to workmen, and especially to labourers, in various countries does not necessarily in any way cheapen the cost of the works which they may undertake. An English workman gets much higher wages than the foreigner, but the amount of work done by the former used to be infinitely greater than that accomplished by the latter within a given number of hours. The main reason assigned for this has been the differences in food, in physique, and in the powers of endurance of the various workpeople. In recent years this same question of food has occupied a considerable amount of attention, especially in the United States, where many instances have occurred of large employers making provision for the food supply of their workpeople, both men and women, and even for the amusement of both, on the ground that the better the health and spirits of the workpeople, the better was the quality of the work done, and the larger the output in the factories and workshops. Pullmantown in the United States is a notable exemplification of this system, where in a large community of thousands of citizens no policeman is required, and there is no public-house. Such results tend to prove that an experiment like that conducted on a large scale by Sir Thomas Lipton through the Alexandra Trust may have a great economic value by improving the physique and the powers of work of the classes, who are dependent for their living upon the work which they undertake to do, and the quality of that work. No doubt much emphasis has been given to the plea that the foreign workman in many trades is able to beat the English workman because of the superiority of his technical education. Whatever force there may be in this contention, it is undoubtedly true, too, that if the English workmen, and especially those engaged in the heavier trades, are to hold their own, the quality and kind of food which they habitually take must be improved, and also the quantity in many cases. For these reasons the Alexandra Trust dining-rooms are calculated to be of high economic value apart altogether from the interest which they must excite amongst Londoners especially, and the true spirit of philanthropy to which their origin is due.

